# Changes in prescribing of psychotropic vs some physical health medication in primary care through the COVID-19 pandemic in England: a national-level survey

**DOI:** 10.1186/s40545-023-00655-9

**Published:** 2023-12-21

**Authors:** Unaiza Waheed, Mike Stedman, Mark Davies, Emma Solomon, David Taylor, Adrian Heald, Ram Prakash Narayanan, John Warner-Levy

**Affiliations:** 1 Department of Diabetes and Endocrinology, Salford Royal Hospital, Salford, M6 8HD UK; 2 The School of Medicine and Manchester Academic Health Sciences Centre, University of Manchester, Manchester, UK; 3 Res Consortium, Andover, UK; 4 Department of Clinical Psychology, Salford Royal Hospital, Salford, UK; 5 Institute of Psychiatry, London, UK; 6 St Helens and Institute of Ageing and Chronic Disease, University of Liverpool, Liverpool, UK

**Keywords:** Psychotropic, Prescribing, Trend, COVID-19, Pandemic

## Abstract

**Introduction:**

The COVID-19 pandemic globally impacted healthcare provision. Prescribing changes in common medications can be used as a marker for new diagnoses. We describe how the prescribing of specific psychotropics was impacted by the pandemic.

**Methods:**

Primary Care Prescribing data for different classes of drugs from March 2017 to February 2022 were considered. To capture the impact during periods of restricted access to health services for new diagnoses/existing conditions, repeat prescriptions/episodic prescribing were included with account taken of historical trends.

The pre-pandemic prescriptions issued each month from March 2018 to February 2020 were linearly extrapolated forward to give an expected annual growth (EAG). The monthly average expected prescriptions for the pandemic period (March 2020–February 2022) were compared.

**Results:**

Physical health medications had lower monthly prescriptions during the pandemic, most markedly for antibiotics − 12.5% (EAG − 1.3%). Bronchodilator prescribing showed a marked increase in the early pandemic months from March 2020 of 5% (EAG 0.1%). Mental health medication prescribing increased above trend for hypnotics/anxiolytics by 0.2% (EAG − 2.3%), while antidepressants fell by − 0.2% (EAG 5.0%), with no net change for antipsychotics (EAG 2.8%), but a temporary increase in antipsychotic prescribing in the early pandemic period. For all the main antidepressants prescribed in England (Sertraline, Mirtazapine, Venlafaxine, Fluoxetine and Citalopram), prescribing actually decreased in the main pandemic period vs historical trend.

**Conclusions:**

The increase in anxiolytic/hypnotic prescribing above trend links to pandemic effects on anxiety/worry. If anything, there was a slight fall in prescribing of the main antidepressants prescribed, which given prevailing circumstances at the time, suggests that access to services may have restricted access to timely assessment.

## Background

First appearing in late 2019, Severe acute respiratory syndrome coronavirus 2 (SARS-CoV-2) is the causative pathogen of Coronavirus disease (COVID-19), and the COVID-19 pandemic [1]. Although the exact origin of COVID-19 is debated, in 2007, Cheng CC et al. forewarned that “the large reservoir of SARS-CoV-like viruses in horseshoe bats, together with the culture of eating exotic mammals in southern China, is a time bomb” [2]. The WHO approximated 14.9 million excess deaths as a result of this global crisis [3]. Although approaches to controlling the virus differed between countries [4], as national lockdowns became widespread worldwide, healthcare provision in relation to both physical and mental healthcare demands was greatly impacted [5, 6], with the effects still being felt globally.

In this study, our aim was to determine how psychotropic prescribing in England in Primary Care was affected by the COVID-19 pandemic, and in addition, compare this to specific other medication groups used to treat physical disorders. Changes in prescribing of commonly used medication can be used as a marker for both diagnoses of new patients and levels of service for existing patients in Primary Care [7, 8].

Although a number of publications have described the consequences of the COVID-19 pandemic in relation to the incidence of depression and other mental health issues [9], the matter of how much the prescribing of specific psychotropic medications was impacted by the pandemic and the tectonic changes in the way that millions of people in developed healthcare systems interacted with their general practices have been less explored.

In relation to this question, we here describe an analysis of prescribing data for all of England in the time before, during and after the main impact period of the COVID-19 pandemic. Making this topic even more relevant is the seeming mental health crisis that was born from this pandemic, whether that be from the direct impact of the virus on mental health itself, or from more indirect and insidious causes, such as the loss of loved ones and feelings of isolation brought on from lockdowns [4]. However, it was reported by Bourmistrova et al., that although SARS-CoV-2 infection was associated with deteriorating mental health in the short term, the long-term prevalence of psychiatric illnesses, including anxiety, depressive disorders, post-traumatic stress disorder (PTSD) and sleep disturbance, is not significantly different to that of the general population [10].

## Methods

Prescription Cost Analysis (PCA) is a National Statistic in England, providing details of the total number of items and the Net Ingredient Cost (NIC) of all prescriptions dispensed in the community. Data from 2017 to 2022 (6 years) was downloaded by BNF section. Medications were broken down by the British National Formulary (BNF) into 15 chapters and 105 sections. Chapter 4 central nervous system includes three sections which are taken as psychotropic 4.01 hypnotics and anxiolytics, 4.02 drugs used in psychoses and related disorders and 4.03 Antidepressants [11].

Medications that were on repeat prescriptions and those with episodic patterns were included. The percent change between the start and end year showed how psychotropic medication had performed compared to total medications.

Different classes of medication are growing and falling at different rates and account was taken of this in our analysis, regarding how the trends in prescribing of psychotropic vs physical medication might differ, to capture the impact during periods of restriction of access to health services for new diagnoses/existing conditions.

A selection of physical and mental health medication British National Formulary (BNF) code classes were selected and the number of prescriptions issued each month in Primary Care was downloaded from the English Prescribing data set [12]. Calculation of the monthly rolling total for the previous 12 months was taken to remove short-term fluctuation effects.

The rolling annual total pre-pandemic prescriptions issued each month from March 2018 to Feb 2020, were linearly extrapolated forward to give an expected annual growth (EAG) and then provide estimates over the pandemic period.

From this, the monthly average expected prescriptions for the pandemic period Mar 2020–Feb 2022 were calculated and compared to the actual average.

To evaluate in more detail the impact on anti-depressants, the same evaluation was carried out on the top six prescribed medications.

Ethics approval was not sought as the analysis used publically available aggregated data.

## Results

The medication part of Prescription Cost Analysis (PCA) showed in 2022 a total of 1.12 billion prescriptions at net ingredient costs of £8.83 billion were issued within Primary Care in England and these have increased 5.0% in number and 7.5% in costs in the 5 years since 2017. These three psychotropic sections had 112 million prescriptions issued (10.1% of total medication).

Table 1 shows the top 25 BNF classes by number of prescriptions and anti-depressants have increased by 26% to become the most prescribed class in 2022.

**Table 1:** Top 25 BNF sections by number of Items

BNF_SECTION CODE and NAME		BNF_CHAPTER CODE and NAME		Number of primary care prescriptions items		% Total	Growth 2017–22
				2017	2022		vs 2017
4.03**	Antidepressant drugs	4	Central Nervous System	67,530,457	85,404,864	8%	26%
2.12*	Lipid-regulating drugs	2	Cardiovascular System	72,612,421	82,961,403	7%	14%
2.05*	Hypertension and heart failure	2	Cardiovascular System	71,531,001	74,707,763	7%	4%
1.03	Antisecretory drugs and mucosal protectants	1	Gastro-Intestinal System	64,699,342	74,241,142	7%	15%
6.01	Drugs used in diabetes	6	Endocrine System	53,009,892	62,740,737	6%	18%
4.07	Analgesics	4	Central Nervous System	65,812,796	60,153,130	5%	− 9%
2.06	Nitrates, calcium-channel blockers and other antianginal drugs	2	Cardiovascular System	49,365,164	54,724,021	5%	11%
2.04*	Beta-adrenoceptor blocking drugs	2	Cardiovascular System	37,816,699	41,569,315	4%	10%
5.01*	Antibacterial drugs	5	Infections	37,060,004	35,786,987	3%	− 3%
6.02	Thyroid and antithyroid drugs	6	Endocrine System	32,170,037	34,210,414	3%	6%
2.09	Antiplatelet drugs	2	Cardiovascular System	35,082,373	32,203,313	3%	− 8%
9.06	Vitamins	9	Nutrition and Blood	30,701,877	32,501,986	3%	6%
2.02	Diuretics	2	Cardiovascular System	33,353,502	29,764,341	3%	− 11%
4.08	Antiepileptic drugs	4	Central Nervous System	26,649,294	31,218,324	3%	17%
3.01*	Bronchodilators	3	Respiratory System	31,228,824	30,381,115	3%	− 3%
3.02	Corticosteroids (respiratory)	3	Respiratory System	20,838,731	22,183,293	2%	6%
10.01	Drugs in rheumatic diseases and gout	10	Musculoskeletal and Joint Diseases	22,805,192	22,047,747	2%	− 3%
9.01	Anaemias and some other blood disorders	9	Nutrition and Blood	18,787,441	22,064,737	2%	17%
7.04	Drugs for genito-urinary disorders	7	Obstetrics, Gynaecology and Urinary-Tract Disorders	19,131,927	21,753,878	2%	14%
2.08	Anticoagulants and protamine	2	Cardiovascular System	16,999,802	20,378,949	2%	20%
1.06	Laxatives	1	Gastro-Intestinal System	18,512,759	18,802,212	2%	2%
3.04	Antihistamines and allergic emergencies	3	Respiratory System	14,359,226	14,608,098	1%	2%
4.01**	Hypnotics and anxiolytics	4	Central Nervous System	15,391,217	13,815,713	1%	− 10%
4.02**	Drugs used in psychoses and related disorders	4	Central Nervous System	11,803,257	13,315,874	1%	13%
14.04	Vaccines and antisera	14	Immunological and Vaccines	13,315,581	11,855,596	1%	− 11%
						of total	
25	Top Subtotal			813,038,359	857,990,088	84.8%	6%
105	Total Medication			1,060,112,747	1,112,920,677		5%
3	Total psychotropic**			94,724,931	112,536,451	10.1%	19%
5	Total Comparators*			250,248,949	265,406,583	23.8%	6.1%

**psychotropic, *comparator groups

**Fig. 1:**
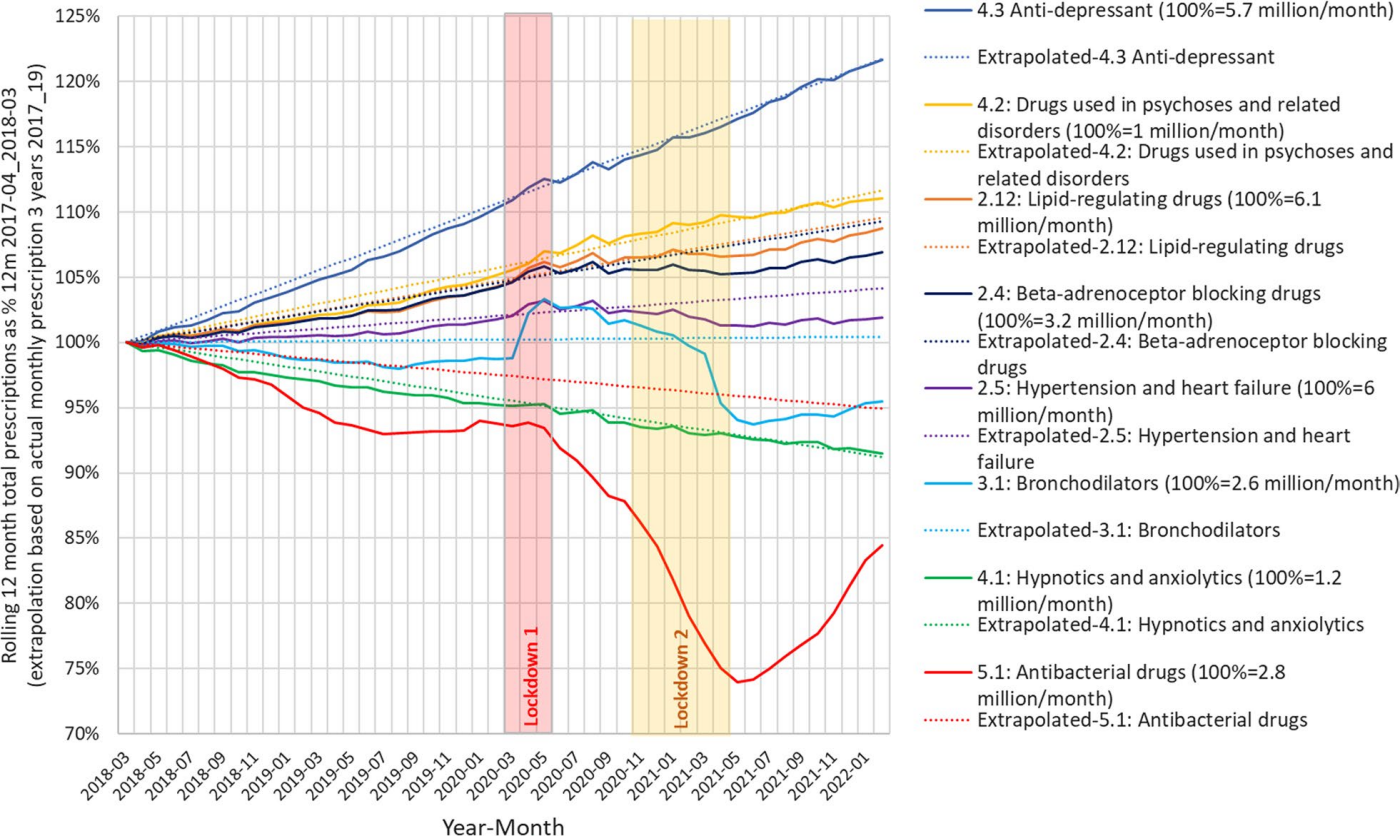
Relative development in Primary Care prescribing mental and physical health medication before and over the pandemic. Shows a rolling previous 12 month total prescriptions by medication class for each month, against the linear extrapolation based on the previous 3 years 2017 to 2019. Results are standardised to the values for the year April 2017_March 18. The time intervals of the main England lockdowns are shown

Figure 1 shows the rolling monthly trend for major physical health and mental health classes the number reflects the BNF class and the 100% = figure represents the monthly average prescription over the initial 12-month period, i.e., approximates to the number of patients being treated. The coloured sections reflect the periods when social restrictions were enforced during the COVID-19 pandemic—this included access to Primary Care for face-to-face appointments.

The following prescribing trends for BNF classes are based on the assumption that for long-term conditions patients are receiving one prescription per month;

Antidepressants started with around 5.7 million people on therapy and decreased slightly over the main pandemic period. Drugs for psychoses started with 1 million people on therapy, increased slightly during the social restrictions but have fallen back onto the trend. Hypnotics and Anxiolytics started with 1.2 million on therapy (decreasing EAG), grew slightly during lockdowns and have now returned to the trend. Lipid-regulating drugs started with 6.1 million on therapy, increased slightly over lockdown but have fallen back. Beta blockers started with 3.2 million on therapy increased during the first lockdown and have since then stopped growing. ACEI and ARBs (Hypertension and Heart Failure) with 6 million on therapy increased over the lockdowns and have since then declined. Bronchodilators started with 2.6 million on therapy, increased sharply over the 1st lockdown and then fell sharply in the 2nd lockdown. Antibiotics started with 2.8 million on therapy (falling EAG) due to the ongoing national antibiotic stewardship program to reduce unnecessary prescribing, fell sharply during the pandemic but are now recovering.

Table 2 compares the actual mental and physical health prescribing to the extrapolation of historical trends to show what might have been expected if there had been no pandemic and the difference to the actual average over the 24-month pandemic main period as a % of the expected prescribing.

**Table 2:** Actual average prescriptions/month over the pandemic 2020 and 2021 were compared to the expected based on extrapolation of 2017 to 2019 giving an expected annual growth (EAG) for each class

	Expected annual growth (EAG) %	Expected average prescription/month	Actual average prescription/month	Difference in number patients on medication	% Difference
4.1: Hypnotics and anxiolytics	− 2.3	1,156,294	1,158,535	2242	0.2
4.2: Drugs used in psychoses and related disorders	2.8	1,061,061	1,061,492	431	0.0
4.3 Anti-depressant	5.0	6,747,420	6,733,576	− 13,844	− 0.2
2.12: Lipid-regulating drugs	2.3	6,608,557	6,575,227	− 33,330	− 0.5
2.5: Hypertension and heart failure	1.0	6,204,487	6,098,043	− 106,444	− 1.7
3.1: Bronchodilators	0.1	2,597,517	2,540,615	− 56,902	− 2.2
5.1: Antibacterial drugs	− 1.3	2,616,588	2,288,535	− 328,053	− 12.5

The difference reflects the difference between actual and expected prescriptions

Compared to declines in physical health prescribing, mental health prescribing of hypnotics/anxiolytics increased by 0.2% above the trend in 2020 and 2021. There was a slight fall in antidepressant prescribing (− 0.2%) in the same period. Antipsychotic prescribing overall kept on-trend.

Physical health medications had lower monthly prescriptions during the pandemic most markedly for antibiotics − 12.5% (EAG − 1.3%). Bronchodilator prescribing showed a marked increase in the early pandemic period from March 2020 of 5% (EAG 0.1%).

**Fig. 2:**
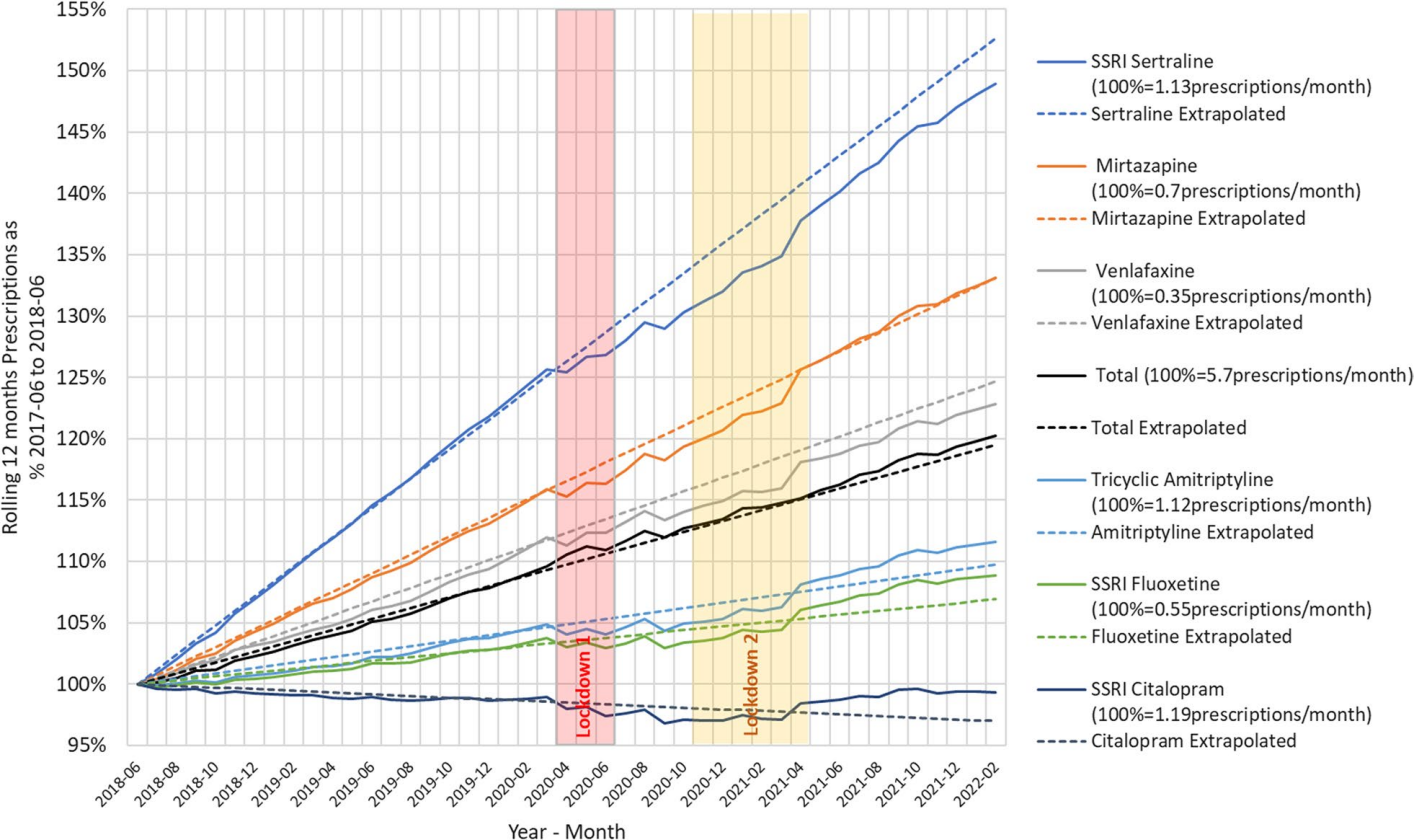
Relative development of prescription for the top six antidepressant agents over the pandemic period. Results are standardised to the values for the year June 2017_May 18. The time intervals of the main England lockdowns are shown

The 6 main antidepressant medications which together take up 88% of total anti-depressant prescriptions were investigated and results are shown in Fig. 2.

The following trends for individual antidepressants are based on the assumption that patients are receiving one prescription per month;

SSRI-Sertraline was used by 1.1 million individuals and was the fastest-growing pre-pandemic. The growth rate reduced during the lockdown and has not recovered to expected levels. Mirtazapine was used by 700,000 people and growth fell during the lockdown but has now recovered back to expected levels. Venlafaxine was used by 350,000 people and the growth rate was reduced during the lockdown and has not recovered to expected levels. Tricyclic Amitriptyline was used by 1.1 million individuals and growth was reduced in lockdown but has now recovered higher than could be expected. SSRI Fluoxetine was used by 550,000 million people and growth was reduced slightly in lockdown but has now recovered higher than could be expected. SSRI Citalopram was used by 1.2 million individuals and was declining before the pandemic fell slightly during lockdown but has now recovered back to higher than could be expected.

Thus for all the main antidepressants prescribed in England (Sertraline, Mirtazapine, Venlafaxine, Fluoxetine and Citalopram), prescribing actually decreased in the main pandemic period vs historical trend.

Net absolute changes for the three main antidepressants over the period analysed were: Sertraline grew by 21% so its share of total anti-depressants increased from 22.7% to 24.6% (+ 8%); Mirtazapine grew by 16% so share increased from 13.0% to 13.6% (+ 5%); Venlafaxine grew by 11% so share stayed at 6.3% (0%).

## Discussion

The increase in anxiolytic/hypnotic prescribing above (Table 1 and Fig. 1) may reflect the impact of the COVID-19 pandemic on the levels of anxiety and worry experienced by many individuals. This observation is supported by the findings of Jacob et al. [13] who reported an increase in the number of patients newly diagnosed with anxiety disorder and Estela et al. [8] who described an increasing trend throughout the pandemic in the prescription of anxiolytics, sedatives, and hypnotics. We suggest that this reflects the experience of many people who have lived through the COVID-19 pandemic.

The increased impact of the COVID-19 pandemic on mental health rather than physical health has been well-described [9]. However, there was no increase in antidepressant prescribing above trend (if anything there was a slight fall in prescribing of the main antidepressants prescribed for much of the main pandemic period) (Fig. 2), which given prevailing circumstances at the time, suggests that access to services may have restricted access to assessment [4]. This is supported by Goyal et al., who also reported that instead of being seen by a healthcare professional, patients seeking a consultation for breathlessness were only given automated safety advice [14]. The fall in antibiotic prescribing in addition to the trend, reflects a combination of reduced access to services [15], particularly face-to-face general practice consultations, limited episodic prescribing, and also the wearing of face masks [10] which would have reduced the transmission of respiratory pathogens. Finally, the precipitate increase in bronchodilator prescriptions at the beginning of the COVID-19 pandemic likely reflects the high level of population anxiety re. becoming seriously unwell with acute COVID-19 prevalent at that time [11], in the first of all England lockdowns.

There was an increase in antipsychotic prescribing in the early phase of the pandemic which may relate to the use of antipsychotics to manage behavioural challenges in people with cognitive impairment in residential care home settings [12]. Specifically, the authors of this paper reported that the proportion of patients with dementia who were prescribed antipsychotics through 2020 substantially increased compared to the years immediately prior to 2020, when the proportion of patients with dementia who were prescribed antipsychotics had tended to be constant. This is evidenced by Yan et al., who reported that in the U.S, antipsychotic use increased among nursing home residents, especially those who are of a minority background [16].

The increase in prescriptions of Sertraline and Mirtazapine over time likely reflects the individual preference of general practitioners as there is no specific recommendation for their use vs other agents in BNF/NICE guidance [13]. The antidepressant prescribing practice seen here is not in keeping with NICE Guidelines with respect to the increased use of Venlafaxine—a 3rd line drug in current and previous guidance. The use of Amitriptyline is, of course, most likely to be for chronic pain rather than depression [17]. The popularity of Mirtazapine is surprising given its sedative effects and propensity for weight gain in individuals who take it regularly. However, general practitioners may also prefer mirtazapine when treating patients with psychiatric disturbance and co-morbid insomnia, due to these sedative effects [18].

Going forward, a person and clinician focussed evaluation of the individual experience of the pandemic in relation to how access to general practice has influenced the prescription of antidepressants and anxiolytics may illuminate why we observe these prescribing trends. This is anticipated to be the next step of our work.

### Strengths/limitations

We have been able to access national-level aggregated data for this analysis. We accept that this does not take into account differences between individual general practices in relation to access to services, nor does it take into demographic factors in relation to access to care and incident mental health issues during the COVID-19 pandemic. We also accept that our analysis cannot differentiate new from repeat prescriptions.

## Conclusions

The increase in anxiolytic/hypnotic prescribing above trend links to pandemic effects on anxiety/worry. If anything there was a slight fall in prescribing of the main antidepressants prescribed, which given prevailing circumstances at the time, suggests that access to services may have restricted access to timely assessment. The increased bronchodilator prescribing in the early pandemic period likely reflects concerns in asthma sufferers regarding the potential effects of COVID-19 infection.

## Data Availability

The data that support the findings of this study are available from the corresponding author upon reasonable request.

## Abbreviations

EAG: Expected annual growth
COVID-19: SARS-CoV-2
BNF: British National Formulary
SSRI: Selective serotonin reuptake inhibitor
NICE: National Institute for Health and Care Excellence
